# Climatic, geomorphologic and hydrologic perturbations as drivers for mid‐ to late Holocene development of ice‐wedge polygons in the western Canadian Arctic

**DOI:** 10.1002/ppp.1977

**Published:** 2018-07-02

**Authors:** J. Wolter, H. Lantuit, S. Wetterich, J. Rethemeyer, M. Fritz

**Affiliations:** ^1^ Alfred Wegener Institute Helmholtz Centre for Polar and Marine Research Research Unit Potsdam, Periglacial Research Section Potsdam Germany; ^2^ University of Potsdam Institute of Earth and Environmental Sciences Potsdam Germany; ^3^ University of Cologne Institute for Geology and Mineralogy Cologne Germany

**Keywords:** carbon, lowland coasts, permafrost degradation, plant macrofossil analysis, tundra vegetation, western Canadian Arctic

## Abstract

Ice‐wedge polygons are widespread periglacial features and influence landscape hydrology and carbon storage. The influence of climate and topography on polygon development is not entirely clear, however, giving high uncertainties to projections of permafrost development. We studied the mid‐ to late Holocene development of modern ice‐wedge polygon sites to explore drivers of change and reasons for long‐term stability. We analyzed organic carbon, total nitrogen, stable carbon isotopes, grain size composition and plant macrofossils in six cores from three polygons. We found that all sites developed from aquatic to wetland conditions. In the mid‐Holocene, shallow lakes and partly submerged ice‐wedge polygons existed at the studied sites. An erosional hiatus of ca 5000 years followed, and ice‐wedge polygons re‐initiated within the last millennium. Ice‐wedge melt and surface drying during the last century were linked to climatic warming. The influence of climate on ice‐wedge polygon development was outweighed by geomorphology during most of the late Holocene. Recent warming, however, caused ice‐wedge degradation at all sites. Our study showed that where waterlogged ground was maintained, low‐centered polygons persisted for millennia. Ice‐wedge melt and increased drainage through geomorphic disturbance, however, triggered conversion into high‐centered polygons and may lead to self‐enhancing degradation under continued warming.

## 1 INTRODUCTION

Ice‐wedge polygons are among the most common periglacial landforms in Arctic lowlands, and abundant features of the Yukon Coastal Plain. During times of peat accumulation, ice‐wedge polygons act as considerable sinks in the global carbon cycle.^1^, ^2^ Widespread degradation and erosion of this peat may thus cause large‐scale changes in carbon cycling.^1^, ^3^ Changes to ice‐wedge polygon morphology may also affect landscape hydrology, depending on polygon type. Polygon rims may provide barriers for surface and subsurface drainage through the active layer (low‐centered polygons), while deeply thawed ice‐wedge troughs may promote flow through interconnected pathways (high‐centered polygons).^4^

Large‐scale climate trends may trigger growth or degradation of ice‐wedge polygons at the regional scale. Widespread permafrost degradation was, for example, recorded during the early Holocene thermal maximum in the western Canadian Arctic.^5^, ^6^, ^7^, ^8^, ^9^ Geomorphological processes triggered by lake drainage or sea level rise may, however, affect topography and surface hydrology on a local to subregional level. This may cause polygon growth or degradation independently of the regional climate trend.^10^, ^11^, ^12^ The respective influence of climate and geomorphology on the evolution of different types of ice‐wedge polygons is not well understood because of large temporal and spatial discrepancies between climatic forcing and geomorphological response processes. In this study we therefore investigated past landscape dynamics on millennial time‐scales to discriminate climate‐driven and geomorphology‐driven changes on ice‐wedge polygon development.

We addressed the spatial heterogeneity^13^, ^14^ within individual ice‐wedge polygons, by applying a multi‐proxy approach, studying six peat cores from three different ice‐wedge polygons, each with one core from the polygon center and one core from the polygon rim or margin. We addressed the following specific research aims:

1. Reconstruction of ice‐wedge polygon development on the Yukon Coastal Plain during the mid‐ to late Holocene.
2. Identification of drivers triggering (i) initiation of ice‐wedge polygon development and (ii) conversion of low‐centered polygons into high‐centered polygons.
3. Determination of factors promoting stability in ice‐wedge polygons.

## 2 BACKGROUND

Ice‐wedge polygons are most widespread in regions underlain by continuous permafrost.^15^, ^16^ They develop in areas with a very low relief energy, where drainage is impeded and the ground stays permanently waterlogged.^17^ They are characterized by wedge‐shaped ice in the ground, which builds up over decades to millennia through repeated thermal contraction cracking during winter and meltwater infiltration into the cracks in spring.^18^ We are using the term ice‐wedge polygon in the sense of polygonal peatlands, i.e., peat‐forming areas underlain by a network of ice wedges that show a surface expression in the form of raised rims and/or low‐lying troughs.

One way to classify different morphological types of ice‐wedge polygons is to distinguish low‐centered polygons from high‐centered polygons.^15^, ^19^ Low‐centered polygons are characterized by raised rims on either side of polygonally adjoining ice wedges enclosing a central depression. Surface flow is impeded, yet not completely prevented, where this type prevails. High‐centered polygons are thought to develop from low‐centered polygons due to (i) improved drainage causing (melt) water flow and thermal erosion along ice wedges, (ii) self‐organization through lateral material displacement as the underlying ice wedges grow wider and rim material is pushed toward the centers of polygons^15^ and/or (iii) increased air temperatures promoting ice‐wedge thaw and wetter polygon troughs.^4^ Relief inversion and an altered landscape hydrological regime result from the conversion.^4^ The raised center consecutively dries up and may be eroded,^20^, ^21^ while thermal erosion along ice wedge pathways may enhance transport of material into adjacent landscapes.

Ice‐wedge formation may be related to large‐scale climate trends. Thermal contraction cracking requires severe ground frost in winter,^19^, ^22^, ^23^, ^24^ which may be provided by a combination of low ambient temperatures and a thin snow cover. Cracking has been shown to be more frequent in peat than in mineral soil.^22^, ^23^ Ice‐wedge polygon development also requires sufficient moisture supply. Ice wedges are fed primarily by hoar formation within cracks in winter and by water from snowmelt and rain in spring. These drivers of ice‐wedge polygon development may, in turn, be influenced by the vegetation cover. In particular, growth height and functional group composition determine effectiveness of ground insulation^25^, ^26^, ^27^, ^28^ and snow retention potential.^29^ Alterations in any of these factors (winter temperatures, snow cover, moisture supply, vegetation composition) may cause changes in cracking frequency or degradation of ground ice, and ultimately trigger changes in ice‐wedge polygon morphology.

Ice‐wedge polygons also experience drastic geomorphological changes, most recently induced by permafrost thaw. Increased thaw has been observed to produce thicker active layers and degrading ice wedges,^4^, ^12^, ^30^, ^31^ while stabilization of deeply degraded ice wedges has been reported to be a result of thermal insulation through the accumulation of organic debris.^30^ Increasing wetness due to increased thaw of ice‐rich permafrost is thought to be reversed in the long run, as increased evapotranspiration during warmer, longer summers is predicted to reduce moisture in the active layer as well as surface water in ponds and lakes.^32^, ^33^, ^34^ Such ambiguous effects acting on various temporal and spatial scales all relate to the interplay between climatic and geomorphological drivers.

Studies of long‐term ice‐wedge polygon development have shown that ice‐wedge polygons may exist in a relatively stable state over millennia.^12^, ^35^, ^36^, ^37^ They are, however, vulnerable toward changes in air temperatures, precipitation and geomorphological disturbance. Recent studies have underlined that ice‐wedge polygons may degrade over the course of years to decades as a response to such changes.^20^, ^30^

## 3 STUDY AREA

The study area is situated on the terrestrial part of the Canadian Beaufort Sea shelf. It is characterized by a subarctic, maritime climate, a flat to slightly undulating topography, and ice‐rich unconsolidated sediments shaped by periglacial processes in the western part and by Pleistocene glaciations superimposed by periglacial processes in the eastern part.^7^

**Figure 1:**
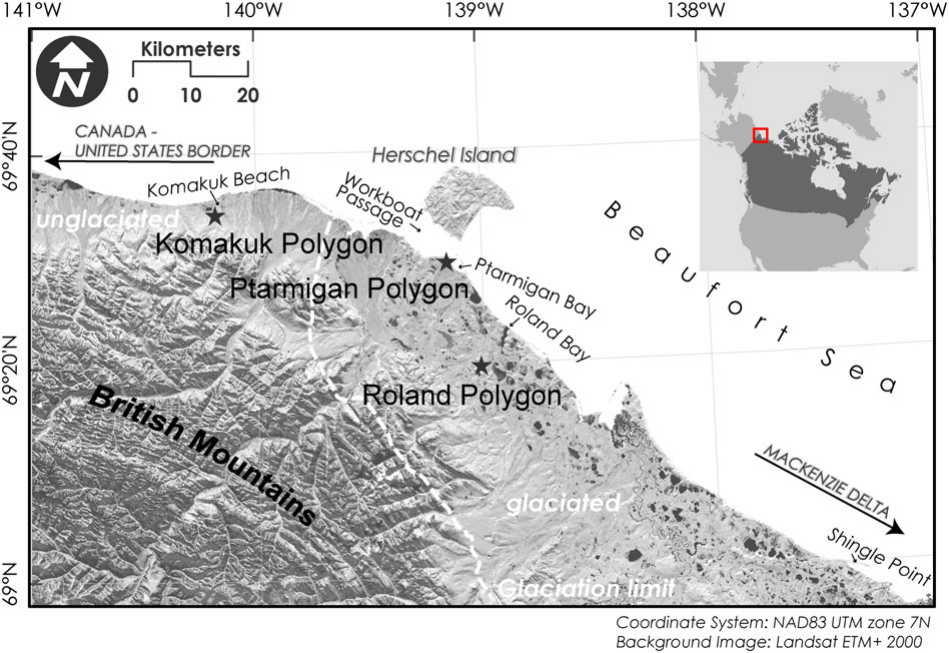
Location of studied ice‐wedge polygons on the Yukon Coastal Plain close to the reconstructed terminal limit of glaciation during the Last Glacial Maximum. Base map modified after Wolter et al.^38^ [Colour figure can be viewed at http://wileyonlinelibrary.com]

The Yukon Coastal Plain stretches across 240 km of coastline from the Mackenzie Delta in the east to the Alaskan border in the west and is bordered by the Beaufort Sea in the north and by the British Mountains in the south, leaving it 10–40 km wide (Figure 1). Situated at about 69°N, the Yukon Coastal Plain has a subarctic climate modified by the Beaufort Sea. Mean annual air temperatures are between −11°C at Komakuk Beach and − 9.9°C at Shingle Point, with respective annual precipitation means of 161.3 and 253.9 mm (1971–2000 means, http://climate. weather. gc.ca). About half of the scarce precipitation falls as snow, resulting in a thin snow cover (mean 25 cm), which is locally variable due to strong wind redistribution and prevails for 250 days per year on average. The topography of the plain is characterized by a flat coastal zone and rolling hills toward the Mountain range. This study focused on the flat coastal reaches, which were shaped by (i) Late Pleistocene advances of the Laurentide Ice Sheet, which reached its furthest extent about 16.2 ka BP^9^, ^39^ and (ii) paraglacial and periglacial processes thereafter. The unglaciated landscape west of about 139.6°W was subject to periglacial conditions throughout the Quaternary, and is characterized by flat, low‐lying wetlands and ice wedge growth. The moraine landscape in the eastern part has higher coastal cliffs composed of thick glacigenic deposits. This leads to large elevation differences between the tops of moraines and the base level of stream erosion and results in relatively deeply incised valleys and generally larger elevation differences than in the unglaciated part. Typical periglacial features on the Yukon Coastal Plain include thermokarst lakes, many of them at least partly drained, ice‐wedge polygons, pingos and retrogressive thaw slumps. Peatland development is favored by continuous permafrost with a shallow active layer (mostly less than 50 cm) and an abundance of low‐lying ground. A permafrost depth of 142 m has been documented near Roland Bay.^40^ The tundra vegetation is dominated by mosses, sedges and dwarf shrubs,^41^, ^42^ with sedges (*Carex* sp) dominating sites with impeded drainage, and tussock cottongrass (Eriophorum vaginatum) dominating better drained, elevated surfaces.^31^ Dwarf shrubs associated with wetlands include various Ericales, *Salix* spp., Betula glandulosa and Rubus chamaemorus, while in river valleys sheltered conditions promote taller growth of the shrubby taxa *Salix* spp., Alnus crispa and Betula glandulosa.^43^

**Figure 2:**
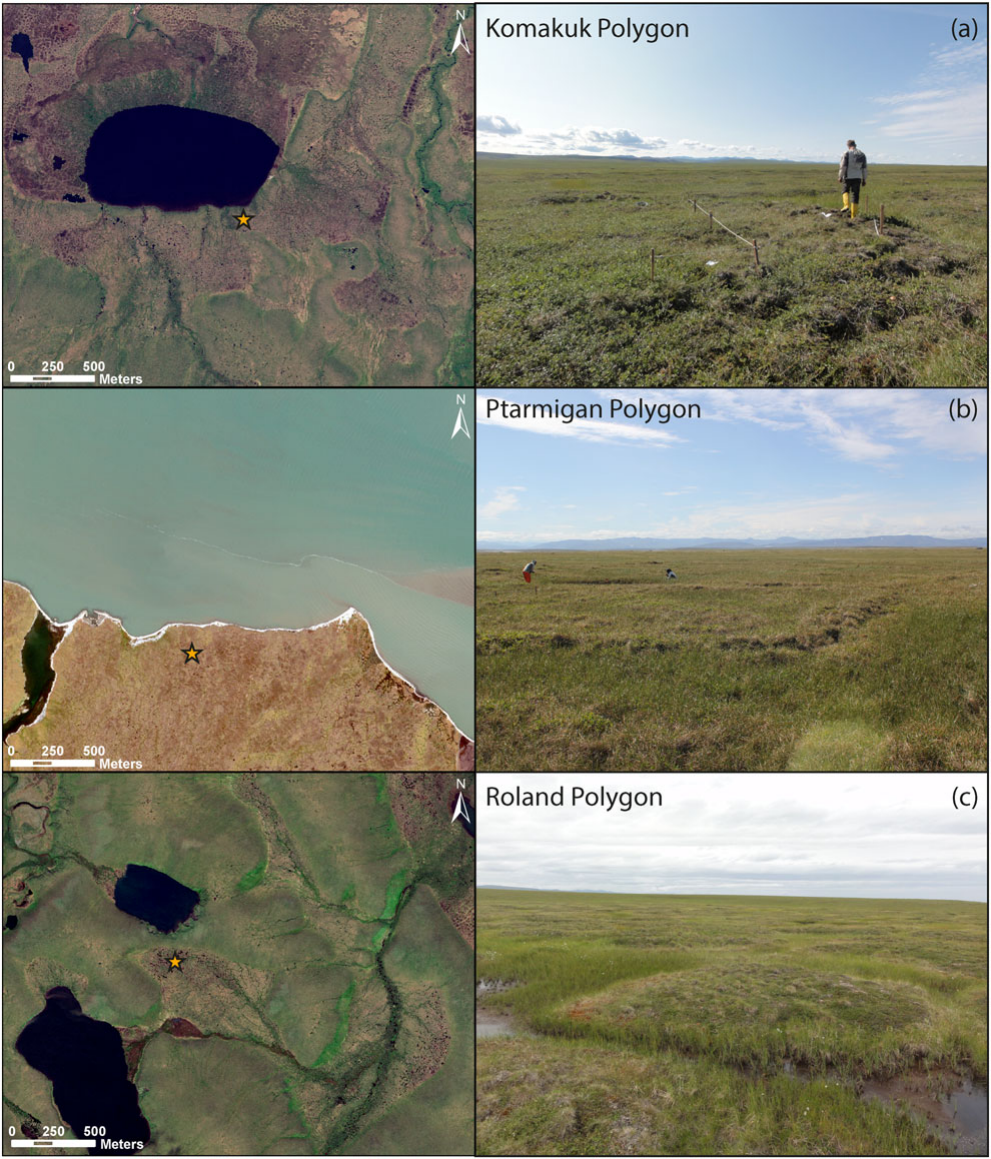
Settings of the studied ice‐wedges polygons. (a) Satellite image and photograph showing surroundings of intermediate‐centered Komakuk Polygon. (b) Satellite image and photograph showing surroundings of low‐centered Ptarmigan Polygon. (c) Satellite image and photograph showing surroundings of high‐centered Roland Polygon. All satellite images are true color pan‐sharpened Geoeye‐1 scenes with 0.5 m ground resolution [Colour figure can be viewed at http://wileyonlinelibrary.com]

We investigated the mid‐ to late Holocene development of three ice‐wedge polygons situated in the western and central coastal reaches of the Yukon (Figures 1, 2). Polygon morphology and vascular plant taxa composition have been summarized in Wolter et al.^31^

Komakuk Polygon (Figure 2a) was formed outside the reconstructed terminal limit of Pleistocene glaciations near Komakuk Beach (69.57959°N, 140.19853°W, Figure 1). The polygon was part of a field of intermediate‐centered polygons on the southern, elevated banks of a lake about 1.5 km from the sea. Komakuk Polygon had a barely discernible raised rim around a slight depression and narrow wet troughs above the surrounding ice wedges. The polygon measured 10 m from rim to rim. The vegetation in the polygon was characterized by taxa typically found on mesic wetland sites, such as Eriophorum vaginatum, and dwarf shrubs including Betula glandulosa, Rubus chamaemorus and Vaccinium vitis‐idaea.

Ptarmigan Polygon (Figure 2b) was situated in a field of degrading low‐centered polygons near Ptarmigan Bay on a glacial outwash plain south of Herschel Island only about 160 m from the coast (69.49979°N, 139.1815°W, Figure 1). It measured 12 × 18 m and had clearly discernible rims enclosing a wet depression that was submerged in places. The polygon was surrounded by water‐filled troughs on three sides and shared the fourth rim with a neighboring polygon. The vascular plant taxa composition showed a clear distinction between low‐lying (mostly *Carex* spp. and *Eriophorum* spp.) and elevated surfaces (*Salix* spp., Dryas integrifolia, Rubus chamaemorus, Pedicularis capitata, Polygonum viviparum, Saxifraga nelsoniana) within the polygon.

Roland Polygon (Figure 2c) was located on a ground moraine between two lakes near Roland Bay and about 8.5 km inland from the coast (69.32471°N, 139.02092°W, Figure 1). It was part of a field of high‐centered polygons and measured 8 × 10 m. Its raised and domed surface was surrounded by water‐filled troughs up to 7 m wide. Vascular plant taxa composition was even across the polygon, and consisted of taxa typical for mesic wetland sites: Betula glandulosa, Salix pulchra, Rubus chamaemorus, Ledum decumbens, Vaccinium vitis‐idaea, *Hierochloë alpina*, Eriophorum vaginatum and Luzula confusa.

## 4 MATERIAL AND METHODS

### 4.1 Field work

Field work at Komakuk Polygon (YC12‐KP) and Roland Polygon (YC12‐RP) was conducted in August 2012, and field work at Ptarmigan Polygon (YC13‐PP) was conducted in July 2013. The sampling approach was identical for all three polygons. The results from a detailed survey of microtopography and vegetation at the sites have been published in Wolter et al.^31^ In the field, we retrieved blocks of 15–20 cm width from the active layer of the ice‐wedge polygons using a saw. In total, we present six such cores, one from the center (labeled as Mc) and one from the margin of each polygon (labeled as Mr), which in the intermediate‐ and low‐centered polygons was represented by the ridge around the polygon. The cores retrieved in 2012 reached depths of between 27 and 33 cm. In Ptarmigan Polygon, an additional permafrost core (PG2161) was drilled directly subjacent to the active‐layer core we retrieved from the polygon center, as the active layer itself was rather shallow (14 cm beneath the ridge and 22 cm beneath the center). The total core length for Ptarmigan Polygon center was 88 cm, including both active layer core and permafrost core. Due to logistical considerations, the permafrost core was photographed, described, and subsampled in 4–5 cm increments in the field before it thawed.

### 4.2 Laboratory analyses

The six active‐layer cores were photographed and described in the laboratory, before being subsampled in 1 cm increments. In three cores (Komakuk Polygon ridge, Roland Polygon centre, Ptarmigan Polygon ridge), the lowermost samples could not be reasonably divided further, so the lowermost 1.5 or 2 cm was taken as one sample. In total, 24 radiocarbon dates were obtained from terrestrial plant macrofossils (Table 1) picked from selected samples. The plant fragments were pretreated with standard acid–alkali–acid (AAA) extraction using 1% HCl (1 h at 60°C plus 10 h at room temperature) and 1% NaOH (4 h at 60°C), which was removed by washing with MilliQ water.^44^ For very small or very fragile samples the extraction time was reduced (1 h, room temperature) or no alkali extraction was applied. AMS radiocarbon dating was carried out in Poznan Radiocarbon Laboratory, Poland (Poz), and CologneAMS, Germany (COL). The results are reported in F^14^C and as conventional radiocarbon ages following the conventions of Stuiver, Polach^45^ and Reimer.^46^ We calibrated the radiocarbon dates using CALIB 7.1 (calibration dataset IntCal13^47^) for pre‐bomb dates and CALIBomb (calibration dataset NHZ1^48^) for post‐bomb dates.

**Table 1:** Results of AMS radiocarbon dating; pretreatment methods include extraction with acid–alkali–acid (AAA) as described in the Methods

Lab code	Depth (cm below surface)	AMS ^14^C radiocarbon age (^14^C y BP)	AMS ^14^C age range Calib 7.1, CALIBomb (>modern dates) (cal y BP) 2 sigma confidence interval	Sample mass (μg C)	F^14^C	Sample pretreatment	Dated material
**Komakuk Polygon**							
**YC12‐KP‐Mr (active layer core from polygon rim)**							
COL2652.1.1	8–9	107 ± 33	12–269	988	0.986 82 ± 0.004 02	AAA	Betula glandulosa twigs and leaf, Cyperaceae leaf, Eriophorum vaginatum seed
Poz#2–56521	15–16	>modern	−60 to −7	430	1.0594 ± 0.0049	A	Ledum decumbens leaf
COL2653.1.1	16–17	4749 ± 40	5327–5588	1000	0.553 64 ± 0.002 77	AAA	Dwarf shrub twigs and bark, Cyperaceae leaves, *Carex* seed
COL2654.1.1	23–24	5031 ± 41	5662–5896	779	0.534 55 ± 0.002 73	A	*Carex* seeds, Cyperaceae leaves, wood
Poz#2–56522	30–31.5	4110 ± 73	4439–4829	763	0.5995 ± 0.0055	AAA	*Dwarf shrub leaves*, *Carex* seeds
**YC12‐KP‐Mc (active layer core from polygon center)**							
COL4945.1.1	9–10	392 ± 40	316–514	504	0.952 42 ± 0.004 74	Short AAA	B. glandulosa and L. decumbens leaves, Cyperaceae leaves, *Eriophorum* and *Carex* seeds
COL4946.1.1	13–14	2553 ± 37	2494–2754	998	0.727 77 ± 0.003 36	Short AAA	*B. glandulosa* leaves, *Carex* seeds
COL4947.1.1	16–17	1562 ± 48	1354–1548	348	0.823 29 ± 0.004 96	Short AAA	Cyperaceae leaves, *Carex* seeds
Poz#2–56519	30–31	1697 ± 25	1546–1693	1008	0.8095 ± 0.0026	AAA	*B. glandulosa* catkin scale, *L. decumbens* leaf, *Carex* seed, Menyanthes trifoliata seed
**Roland Polygon**							
**YC12‐RP‐Mr (active layer core from polygon rim)**							
COL2655.1.1	8–9	42 ± 32	−5 to 256	1000	0.994 79 ± 0.004 02	AAA	*B. glandulosa* leaves and fruit, *L. decumbens* leaves
COL2656.1.1	11–12	124 ± 33	9–274	1000	0.984 65 ± 0.004 02	A	*B. glandulosa* leaves and twigs, dwarf shrub twigs
Poz#2–56550	13–14	>modern	−60 to −6	947	1.0426 ± 0.0032	AAA	*B. glandulosa* and *L. decumbens* leaves
COL2657.1.1	16–17	4426 ± 58	4864–5285	337	0.576 42 ± 0.004 13	AAA	*B. glandulosa* twig, Cyperaceae leaves, *Carex* seeds
COL2658.1.1	18–19	5871 ± 59	6507–6846	296	0.481 51 ± 0.003 53	AAA	Cyperaceae leaves, *Carex* seed
Poz#2–56551	26–27	6192 ± 34	6984–7237	671	0.4626 ± 0.0020	AAA	Ericaceae leaves, *Carex* seeds
**YC12‐RP‐Mc (active layer core from polygon center)**							
COL2659.1.1	11–12	170 ± 36	0–294	558	0.979 12 ± 0.004 43	A	*B. glandulosa* leaves *and fruit*, *L. decumbens* leaves
COL2660.1.1	13–14	185 ± 33	0–301	994	0.977 29 ± 0.004 05	A	*B. glandulosa* leaves *and fruit*, *L. decumbens* leaves, *Carex* seed
COL2661.1.1	20–21	592 ± 33	538–652	1000	0.928 92 ± 0.003 81	AAA	*B. glandulosa* twigs, *L. decumbens* leaf, wood, *Carex* seed
Poz#2–56549	25–26	6147 ± 37	6948–7161	874	0.4652 ± 0.0022	AAA	Ericaceae leaves
**Ptarmigan Polygon**							
**YC13‐PP‐Mr (active layer core from polygon rim)**							
COL2651.1.1	13–15	1199 ± 55	982–1265	291	0.861 38 ± 0.005 89	AAA	Cyperaceae leaves, *Carex* seed
**YC13‐PP‐Mc (active layer core from polygon center)**							
COL4942.1.1	4–5	>modern	−43 to −8	422	1.154 73 ± 0.005 53	Short AAA	Cyperaceae leaves
**PG2161 (permafrost core from polygon center)**							
COL4943.1.1	24–28	>modern	−60 to −7	405	1.054 37 ± 0.005 44	Short AAA	Cyperaceae leaves
COL4944.1.1	56–60	1532 ± 55	1318–1535	226	0.8264 ± 0.005 61	Short AAA	Terrestrial plant remains
COL2650.1.1	83–88	5609 ± 42	6304–6470	913	0.497 ± 0.003	A	Terrestrial plant remains

We measured total organic carbon (TOC) and total nitrogen (TN) on freeze‐dried, ground subsamples using an Elementar Vario Max C analyzer (TOC) and an Elementar Vario EL III analyzer (TN). Element contents are expressed as weight percent (wt.%). The analysis of stable carbon isotopes (δ^13^C) was conducted on freeze‐dried, ground, carbonate‐free subsamples at Helmholtz Centre, Potsdam GFZ German Research Centre for Geosciences, Potsdam, Germany, using a Thermo Fisher Scientific DELTAplusXL mass spectrometer. Stable carbon isotope analyses on subsamples of core YC12‐RP‐Mr were measured at Alfred Wegener Institute Helmholtz Centre for Polar and Marine Research, Potsdam, Germany, using a Finnigan MAT DELTA‐S mass spectrometer. All δ^13^C values are expressed as per mil relative to the Vienna PeeDee Belemnite standard (‰ vs. VPDB). Grain size analyses were carried out on carbonate‐free and organic‐free subsamples using a Beckman Coulter LS 200 laser diffraction particle sizer. In the upper centimeters of the cores, grain size analyses were precluded by very low contents of minerogenic material in the peat. Grain sizes are given as volume percent (vol.%). Plant macrofossil analyses were conducted on selected subsamples (3–11 per core, 46 in total). For each subsample, 50 mL of sediment was wet sieved through 1 mm and 250 μm mesh sizes. Due to the large amounts of coarse organic material in the samples, we limited the analyses to picking and identifying vascular plant remains in the >1 mm fraction. This approach provided an overview of vascular plant taxa that were present in the cores, while a full plant macrofossil analysis would have included smaller seeds.

### 4.3 Data and statistical analyses

The zonation presented for the cores was delineated using the Constrained Incremental Sum of Squares (CONISS) algorithm^49^ in package “rioja”^50^ and validated by broken stick modeling in the software R, version 3.2,^51^ based on the parameters TOC, TOC/TN and δ^13^C. The CONISS algorithm performs cluster analysis with the precondition that only adjacent samples may be clustered. This stratigraphically constrained clustering provides a quantitative multivariate method for establishing zones in a stratigraphic context.^49^ We determined the maximum number of valid zones in each core by comparing the clustering result with the random zone distribution provided by a broken stick model, accepting only the number of zones that explain more variance than the random model (see Bennett^52^ for a full account of the method). Principal component analysis (PCA) aided our interpretation of sediment characteristics, the plant macrofossil record, and our first aim of reconstructing the development of the sites. We performed PCA of plant macrofossil count data using all taxa that occurred in at least two of the polygons. For these seven vascular plant taxa from 40 samples we used the sums of all remains found. Some taxa were very numerous in some samples, while mostly there were 1–10 occurrences per sample. To compensate for this imbalance we used Hellinger‐transformed count data. In a second PCA we assessed all 165 core samples based on sediment data (TOC, TOC/TN, δ^13^C). Sediment data were range‐transformed to ensure the data are on the same scale. PCAs were conducted using the function “princomp” in R. Hellinger and range transformations were executed using the function “tran” in the package *analogue* 53 in R.

## 5 RESULTS

### 5.1 Komakuk Polygon

**Figure 3:**
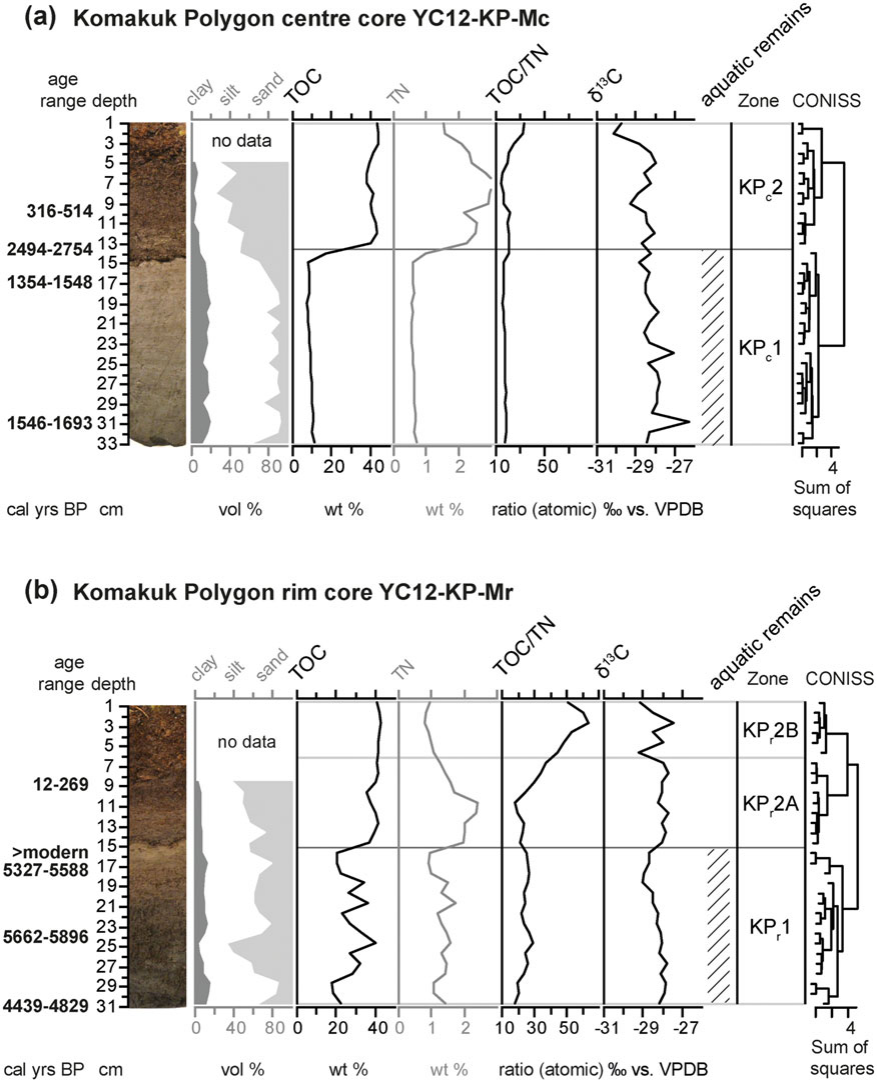
Stratigraphic diagrams showing sediment parameters and established zonation (a) in the center core and (b) in the rim core of Komakuk Polygon. Parameters used in the CONISS analysis are shown in black, while additional parameters not used in this analysis are shown in grey. The age ranges shown are calibrated 2σ ranges based on AMS radiocarbon dates (Table 1). The presence of aquatic organisms in the macrofossil record is indicated by hatching [Colour figure can be viewed at http://wileyonlinelibrary.com]

The active layer core from the center of Komakuk Polygon (YC12‐KP‐Mc) had a median basal age of 1597 cal years BP. The core showed a distinct sedimentary facies break at 14 cm depth, which was accompanied by an age inversion (Table 1, Figure 3a). CONISS ordination validated by broken stick modeling supported two zones KP_c_1 and KP_c_2 for the core, which corresponded with the facies break and were mainly distinguished by a sharp upward increase in TOC (Figure 3a).

In zone KP_c_1 (14–33 cm depth), TOC was low to intermediate, while TOC/TN showed relatively low values and δ^13^C was relatively high (Figure 3a). In KP_c_1, inorganic material was fine‐grained, consisting of clayey silt and sandy silt, with about 20% plant material. A mixture of mesic terrestrial (wood fragments, occasional remains of Betula glandulosa, Ledum decumbens, cf. Ranunculus lapponicus), wet terrestrial (occasional *Carex* sp. seeds) and aquatic (Menyanthes trifoliata, *Potamogeton* sp.) plant macrofossils was preserved in this zone (Table 2).

**Table 2:** Summary of identified vascular plant macrofossils from the center and rim cores of Komakuk Polygon. The overall composition of the organic material is described by giving the amount of plant material after sieving through 1 mm mesh size and the respective estimated amounts of bryophyte, Cyperaceae and wood remains in each sample. Plant macrofossils are ordered by hydrological requirements from taxa found under mesic conditions typical for ice‐wedge polygon rims to taxa found in wet conditions typical for ice‐wedge polygon centers and aquatic plant remains typical for subarctic ponds and lakes

Depth (cm)	Age range (cal y BP)	Amount plant material in sample (mL)	Amount bryophytes in sample (mL)	Amount Cyperaceae in sample (mL)	Amount wood in sample (mL)	*B. glandulosa* twig	*B. glandulosa* leaf (fragment)	*B. glandulosa* fruit	*B. glandulosa* catkin scale	*L. decumbens* leaf	V. vitis‐idaea leaf	E. vaginatum seed	cf R. lapponicus seed	Dwarf shrub twig fragment	Dwarf shrub leaf fragment	*Carex* sp. seed	M. trifoliata seed	P. palustris seed	*Potamogeton* sp. seed	Zone
						Terrestrial											Aquatic			
						Mesic								General		Wet	Emergent		Submerged	
**Komakuk Polygon center core YC12‐KP‐Mc (active layer)**																				
10	316–514	50	12.5	37.5	< 0.1	1	1			1+		3				3				KP_c_2
14	2494–2754	20	0	14	6		2									2				
15		10	0	2	8	1													4	KP_c_1
16		10	0	2	8								1			1			3	
17	1354–1548	10	0.5	7	2.5											3			1	
31	1546–1693	7	0	2.1	4.9	1			1	1						1	1			
32		10	0	4	6	1										1			1	
33		10	0	4	6			1								2			1	
**Komakuk Polygon rim core YC12‐KP‐Mr (active layer)**																				
5		50	30	7.5	12.5	5	4+	1		14+	18+	20				1				KP_r_2B
9	12–269	50	7.5	27.5	15	52	5	1		2	1+	2		> 50		3				KP_r_2A
14		50	0	40	10							2				1				
15		50	0	47.5	2.5														2	
16	>modern	25	0	20	5					1										KP_r_1
17	5327–5588	35	0	31.5	3.5								1	6		2		1		
23		50	0	45	5											12				
24	5662–5896	45	0	27	18									1		14			1	
29		45	0	31.5	13.5	3										43		1		
31	4439–4829	40	0	32	8	4	1			4					2	23			2	

Zone KP_c_2 (0–13 cm depth) uniformly showed very high TOC contents, increasing TOC/TN toward the top of the core and decreasing δ^13^C (Figure 3a). The grain size composition was classified as silty sand. The amount of plant material rose to 100% in this zone (Table 2). Mesic (Betula glandulosa, Ledum decumbens, Eriophorum vaginatum) and wet taxa (*Carex* sp.) were found. Remains of aquatic plant taxa were absent.

The active layer core from the rim of Komakuk Polygon (YC12‐KP‐Mr) showed a hiatus of about 5000 years between 16 and 17 cm depth (Table 1). The identified seeds and leaves of terrestrial plants from the upper part of the core (0–16 cm) showed ages within the last 300 years, while samples below that depth were dated to the middle Holocene (Table 1, Figure 3b), with an age inversion at the base of the core. A sedimentary facies break was evident at 14–15 cm depth, and two stratigraphic zones were delineated on the basis of CONISS ordination and broken stick modeling. In the upper zone, two subzones were identified. The boundary between zones KP_r_1 and KP_r_2 corresponded roughly with the age hiatus.

In zone KP_r_1 (16–31 cm depth) TOC values were intermediate to very high, with a high variability and accompanied by stable TOC/TN values and decreasing δ^13^C values upcore (Figure 3b). Grain size composition fluctuated between sandy silt and silty sand in KP_r_1. Wood fragments and identifiable plant macrofossils were abundant in the zone, especially in the lower part, where seeds of the wet terrestrial *Carex* sp. dominated, accompanied by occasional seeds of the aquatic *Potamogeton* sp. and Potentilla palustris as well as remains of mesic terrestrial Betula glandulosa and Ledum decumbens (Table 2).

Zone KP_r_2 (0–15 cm depth) showed very high and uniform TOC contents, while TOC/TN ratios increased strongly toward the top of the core, and δ^13^C values were stable (Figure 3b). The two subzones were distinguished by an increase in TOC/TN from zone KP_r_2A to zone KP_r_2B. Grain size analyses classified inorganic particles in KP_r_2A as silty sand and sandy silt. Very little inorganic material was present in KP_r_2B, and grain size analyses could not be carried out. Mesic terrestrial taxa (Betula glandulosa, Ledum decumbens, Vaccinium vitis‐idaea, Eriophorum vaginatum) dominated in this zone, while remains of wet terrestrial taxa (*Carex* sp.) were scarce and aquatic taxa (*Potamogeton* sp.) disappeared above 15 cm core depth (Table 2). There was a strong increase in remains of mesic terrestrial taxa from KP_r_2A to KP_r_2B.

### 5.2 Ptarmigan Polygon

**Figure 4:**
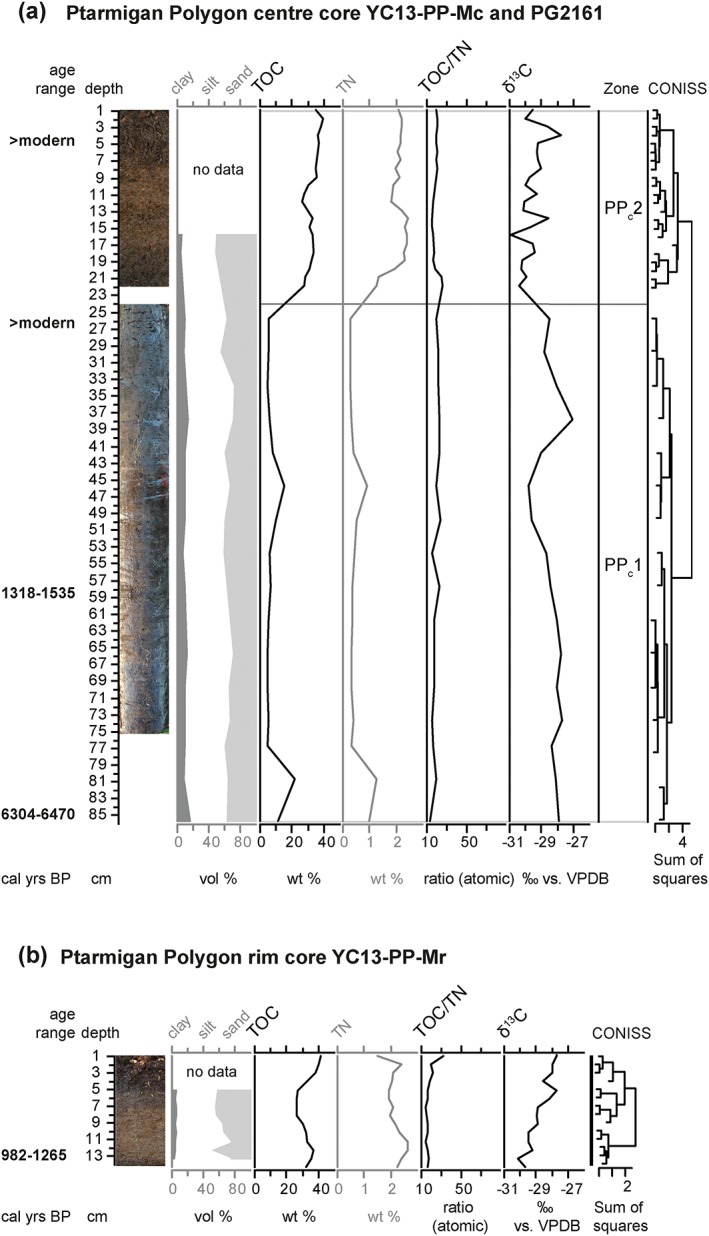
Stratigraphic diagrams showing sediment parameters and established zonation (a) in the center core and (b) in the rim core of Ptarmigan Polygon. Parameters used in the CONISS analysis are shown in black, while additional parameters not used in this analysis are shown in grey. The age ranges shown are calibrated 2σ ranges based on AMS radiocarbon dates (Table 1) [Colour figure can be viewed at http://wileyonlinelibrary.com]

The permafrost core from the center of Ptarmigan Polygon had a median basal age of 6380 cal years BP at 88 cm depth and a median age of 1433 cal years BP at 60 cm depth. The upper parts of the core (at least until 28 cm core depth) had post‐bomb radiocarbon dates (Table 1, Figure 4a). We delineated two stratigraphic zones PP_c_1 and PP_c_2 in the active layer core (YC13‐PP‐Mc) and subjacent permafrost core (PG2161) (Figure 4a).

In zone PP_c_1 (24–86 cm depth) organic matter was characterized by low to intermediate TOC contents, uniformly low TOC/TN ratios and relatively high δ^13^C values (Figure 4a). Zone PP_c_1 had a sandy silt texture. Coring was stopped as a coarse‐grained layer containing gravel was hit. Identifiable plant macrofossils occurred in low numbers (Table 3). In the lower parts of the zone, occasional *Carex* sp. seeds were found. The amount of plant material was generally low, with unidentified plant fragments mostly being Cyperaceae, and very few small fragments of wood and Bryophyte leaflets.

**Table 3:** Summary of identified vascular plant macrofossils from the center and rim cores of Ptarmigan Polygon. The overall composition of the organic material is described by giving the amount of plant material after sieving through 1 mm mesh size and the respective estimated amounts of bryophyte, Cyperaceae and wood remains in each sample. Plant macrofossils are ordered by hydrological requirements from taxa found under mesic conditions typical for ice‐wedge polygon rims to taxa found in wet conditions typical for ice‐wedge polygon centers

Depth (cm)	Age range (cal y BP)	Amount plant material in sample (mL)	Amount bryophytes in sample (mL)	Amount Cyperaceae in sample (mL)	Amount wood in sample (mL)	*B. glandulosa* leaf (fragment)	*L. decumbens* leaf	Dwarf shrub twig fragment	*Carex* sp. seed	Zone
						Terrestrial				
						Mesic		General	Wet	
**Ptarmigan Polygon center core**										
5	>modern	50	0	50	< 0.1			1	1	PP_c_2
20		50	0	50	< 0.1					
28	>modern	5	0.5	2.5	2					PP_c_1
48		10	0.5	9	0.5					
60	1318–1535	5	0	2.5	2.5				1	
75		5	0.5	1.5	3				1	
88	6304–6470									
**Ptarmigan Polygon ridge core**										
3		50	0	35	15	1+	10+	> 50		PP_r_1C
5		40	0	12	28			> 50		PP_r_1B
15	982–1265	50	2.5	47.5	0	1				PP_r_1A

In zone PP_c_2 (0–23 cm depth), organic matter was characterized by high TOC contents, while TOC/TN ratios were similar to those found in zone PP_c_1, and δ^13^C was slightly lower than in PP_c_1 (Figure 4a). The sediment texture was silty sand. In this zone, samples consisted nearly entirely of Cyperaceae peat, yet identifiable plant macrofossils were nearly absent, consisting of one fragment of a dwarf shrub twig and one *Carex* sp. seed (Table 3).

The active layer core from the rim of Ptarmigan Polygon (YC13‐PP‐Mr) had a median basal age of 1127 cal years BP. CONISS ordination validated by broken stick modeling supported no zonation in the core (Figure 4b). TOC values were high, and TOC/TN ratios as well as δ^13^C were intermediate, increasing toward the top of the core (Figure 4b). The texture of the scarce inorganic material was dominated by silty sand. All samples were nearly entirely made up of plant material (Table 3). In the lower part of the core, they consisted of Cyperaceae with a low amount of Bryophytes and one small leaf fragment of Betula glandulosa. In the middle part Cyperaceae and wood, together with abundant dwarf shrub twigs were found. In the upper part, plant material consisted of Cyperaceae and wood, and fragments of at least one Betula glandulosa leaf, Ledum decumbens leaves and abundant fragments of dwarf shrub twigs were found.

### 5.3 Roland Polygon

**Figure 5:**
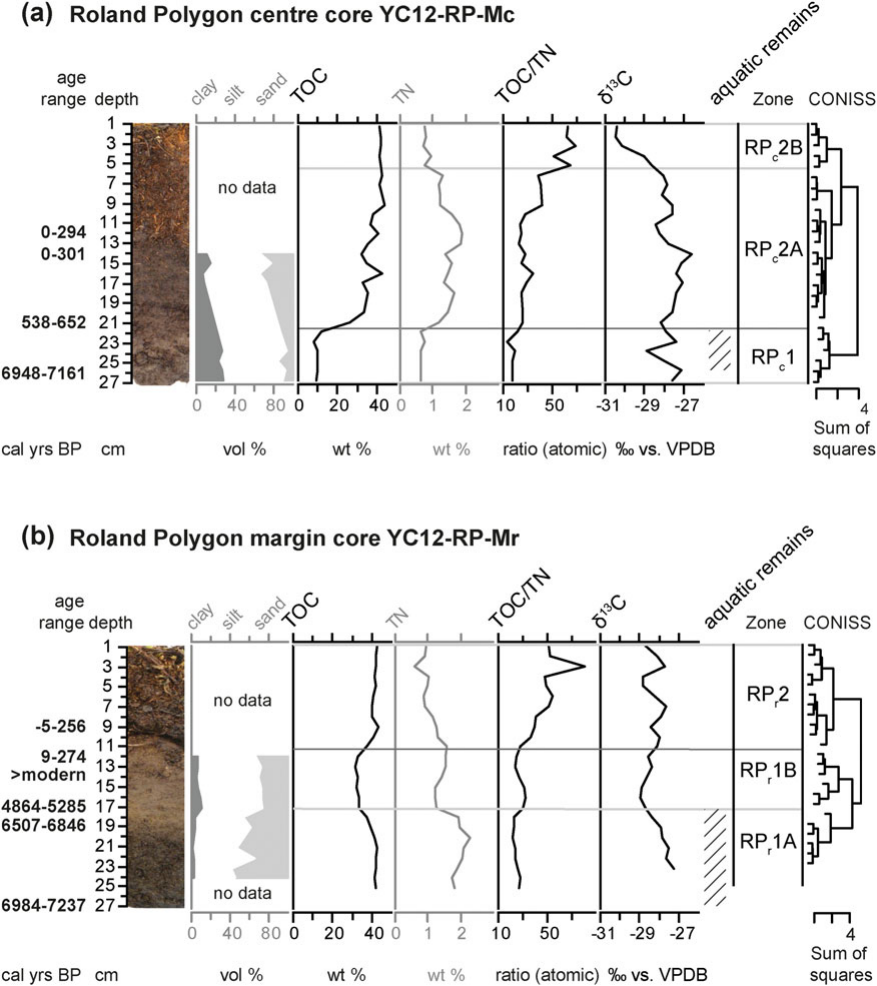
Stratigraphic diagrams showing sediment parameters and established zonation (a) in the center core and (b) in the rim core of Roland Polygon. Parameters used in the CONISS analysis are shown in black, while additional parameters not used in this analysis are shown in grey. The age ranges shown are calibrated 2σ ranges based on AMS radiocarbon dates (Table 1). The presence of aquatic organisms in the macrofossil record is indicated by hatching [Colour figure can be viewed at http://wileyonlinelibrary.com]

The active layer core from the elevated center of Roland Polygon (YC12‐RP‐Mc) showed a hiatus but no age inversions (Table 1). The upper two dated samples at 12 and 14 cm depth originated within the last 300 years. At 21 cm depth, the calibrated median age was 603 cal years BP. A hiatus of nearly 6500 years lay between this sample and the next dated sample at 26 cm depth, which had a median age of 7058 cal years BP. On the basis of CONISS analysis of TOC, TOC/TN and δ^13^C, we established two stratigraphic zones in the core (Figure 5a). The boundary between RP_c_1 and RP_c_2 reflects the hiatus at 21 cm depth and 603 cal years BP. The upper zone RP_c_2 was divided into two subzones RP_c_2A and RP_c_2B.

RP_c_1 (22–27 cm depth) was characterized by low TOC values, low TOC/TN ratios and relatively high δ^13^C values (Figure 5a). The sediment texture in RP_c_1 was clayey silt. Plant macrofossils were abundant and relatively diverse in this zone, with remains of mesic terrestrial dwarf shrubs (Betula glandulosa, Empetrum nigrum, Ledum decumbens) alongside seeds of wet terrestrial *Carex* and emergent aquatic Hippuris vulgaris, Menyanthes trifoliata, and Potentilla palustris (Table 4). Remains of submerged aquatics (*Potamogeton* sp., Charophyta oogonia, *Daphnia* ephippiae) were frequent in this zone.

**Table 4:** Summary of identified vascular plant macrofossils from the center and rim cores of Roland Polygon. The overall composition of the organic material is described by giving the amount of plant material after sieving through 1 mm mesh size and the respective estimated amounts of bryophyte, Cyperaceae and wood remains in each sample. Plant macrofossils are ordered by hydrological requirements from taxa found under mesic conditions typical for ice‐wedge polygon rims to taxa found in wet conditions typical for ice‐wedge polygon centers and aquatic plant remains typical for subarctic ponds and lakes

Depth (cm)	Age range (cal y BP)	Amount plant material in sample (mL)	Amount bryophytes in sample (mL)	Amount Cyperaceae in sample (mL)	Amount wood in sample (mL)	*B. glandulosa* twig	*B. glandulosa* leaf (fragment)	*B. glandulosa* fruit	*B. glandulosa* catkin scale	E. nigrum seed	*L. decumbens* leaf	*V. vitis‐idaea* leaf	*E. vaginatum* seed	Dwarf shrub twig fragment	Dwarf shrub leaf fragment	cf *Luzula* seed	*Carex* sp. seed	H. vulgaris seed	*M. trifoliata* seed	*P. palustris* seed	*Potamogeton* sp. seed	Charophyta oogonia	*Daphnia* sp. ephippiae	Zone
						Terrestrial												Aquatic						
						Mesic								General			Wet	Emergent			Submerged		Animal	
**Roland Polygon center core**																								
6		50	35	12.5	2.50	15+	> 50	21	4		> 50		9	1										RP_c_2B
7		50	20	25	5	12	> 50	2	1		> 50		4				1							RP_c_2A
12	0–294	50	15	32.5	2.50		< 20	1			> 30	1												
14	0–301	50	20	20	10	15	> 50	18	2		> 50		9			1	3							
18		45	13.50	13.5	18	18	< 20	13			> 50		5				2							
21	538–652	25	5	10	10						1						1							
22		25	3.75	10	11.25					1						1	4			1	2	1	3	RP_c_1
24		25	1.25	10	13.75					2	9+						12		2		5	43	21	
25		20	2	10	8						5						4	1		1	3			
26	6948–7161	25	2.50	7.5	15	2				1	2+													
27		20	6	12	2	2				1	2+				1		1							
**Roland Polygon margin core**																								
9	>modern	50	25	10	15	1	> 30	2			9+		10				1							RP_r_2
12	9–274	30	1.50	15	13.50	> 30	> 20	2			3		3				1							RP_r_1B
14	>modern	30	1.50	21	7.50		< 20	5			1		2											
17	4864–5285	40	2	34	4	1							1				11							
19	6507–6846	45	2.25	36	6.75		1				1		1				20						17	RP_r_1A
26		50	25	10	15		1			2	> 50	2					50							
27	6984–7237	50	22.50	22.5	5					9	5+	1			1		30						2	

Zone RP_c_2 (0–21 cm depth) showed high TOC values and a strong increase in TOC/TN, accompanied by a marked decrease in δ^13^C (Figure 5a). The strongest increase in TOC/TN was accompanied by the strongest decrease in δ^13^C from RP_c_2A to RP_c_2B, marking the boundary between the subzones. The grain size analyses classified inorganic particles from RP_c_2 as sandy silt. Mesic terrestrial plant macrofossils (Betula glandulosa, Ledum decumbens, Eriophorum vaginatum) became particularly abundant in RP_c_2, while *Carex* sp. seed occurrence declined gradually and aquatic remains disappeared entirely.

The active layer core from the margin of the high‐centered Roland Polygon (YC12‐RP‐Mr) showed a similar hiatus and had a median basal age of 7085 cal years BP that was nearly identical to the one in the center core YC12‐RP‐Mc from the same polygon (Table 1). The age‐depth relationship was also remarkably similar to the one found in the rim core of Komakuk Polygon. The upper part of the core showed post‐bomb ages or ages of up to 300 cal years BP, and mid‐Holocene ages below 17 cm depth. The core showed no age inversion. A sedimentary facies break was present at 10–11 cm core depth. Two stratigraphic zones RP_r_1 and RP_r_2 were delineated based on CONISS ordination of parameters characterizing organic matter (TOC, TOC/TN, δ^13^C), and the lower zone was divided into subzones RP_r_1A and RP_r_1B (Figure 5b).

RP_r_1 (12–27 cm depth) had high TOC contents, while TOC/TN ratios were intermediate and δ^13^C decreased slightly upcore (Figure 5b). TOC decreased from subzone RP_r_1A to RP_r_1B. The grain size composition changed from silty sand in RP_r_1A to sandy silt in RP_r_1B. Plant macrofossils were abundant in zone RP_r_1, and were dominated by terrestrial taxa (Betula glandulosa, Empetrum nigrum, Ledum decumbens, Vaccinium vitis‐idaea, Eriophorum vaginatum, *Carex* sp.) (Table 4). Remains of Betula glandulosa were rare in RP_r_1A but became abundant in RP_r_1B, while seeds of the wet terrestrial *Carex* sp. were abundant in RP_r_1A, and decreased strongly toward RP_r_1B. The only aquatic indicators were *Daphnia* ephippiae found in RP_r_1A. The trend toward more mesic taxa was mirrored by the occurrence of Eriophorum vaginatum seeds, which were missing from the lower part of RP_r_1A, and increased toward the upper part of RP_r_1B.

In RP_r_2 (0–11 cm depth), TOC contents were high and TOC/TN ratios increased strongly upcore, while δ^13^C was stable (Figure 5b). There was no information on grain size composition for RP_r_2, as the peat contained very little inorganic material. Plant macrofossils were dominated by abundant remains of the mesic terrestrial taxa Betula glandulosa, Ledum decumbens and Eriophorum vaginatum (Table 4).

## 6 DISCUSSION

**Figure 6:**
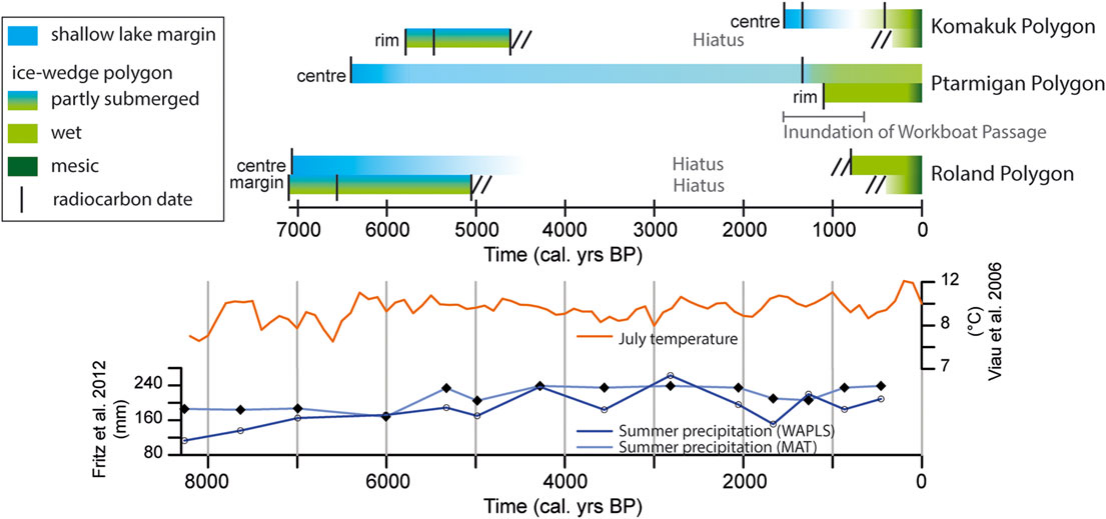
Summary of environmental development in the six cores from three ice‐wedge polygons on the Yukon Coastal Plain. The development from wetter (blue color) to drier (light to dark green color) conditions over the past 7000 years is shown alongside regional climate reconstructions by Viau et al^54^ and Fritz et al.^55^ Median calibrated AMS 14C ages are represented by black vertical lines [Colour figure can be viewed at http://wileyonlinelibrary.com]

The results of sediment and plant macrofossil analyses on the six short cores suggest that all sites experienced change (Figure 6). In the mid‐Holocene wetter conditions prevailed at all sites, which were shallow lakes or submerged ice‐wedge polygons. This was followed by a 5000‐ to 6000‐year hiatus indicating disturbance in the intermediate‐centered Komakuk Polygon and the high‐centered Roland Polygon. The initiation or re‐initiation of ice‐wedge polygon development roughly fell within the last millennium. Finally, all three polygons experienced recent degradation and drying. Low‐lying surfaces were converted into elevated surfaces and the vegetation composition changed markedly (Tables 2, 3, 4).

**Figure 7:**
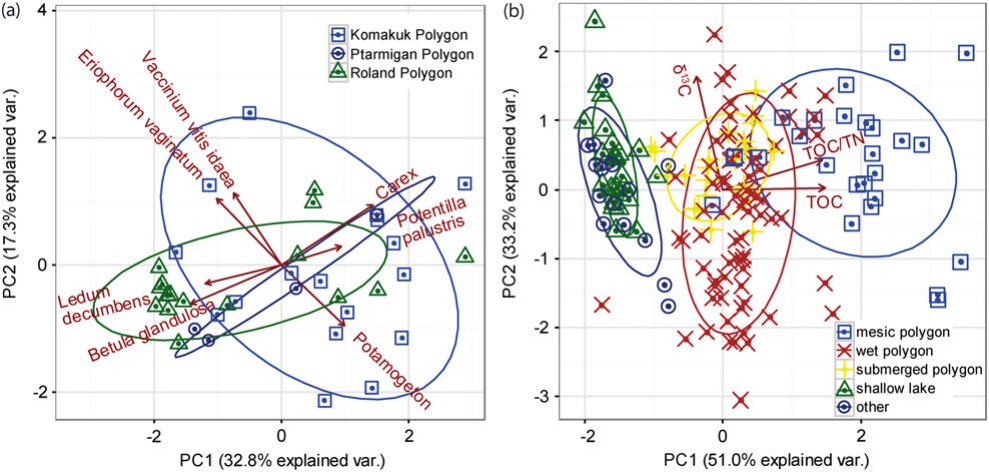
Principal component analysis (PCA) biplots for all cores combined. (a) PCA biplot based on plant macrofossil taxa counts of selected samples; sample scores are identified according to which ice‐wedge polygon site they belong to. (b) PCA biplot based on the sediment parameters total organic carbon (TOC), C/N and δ^13^C from all samples; sample scores are identified according to our environmental interpretation of core sections [Colour figure can be viewed at http://wileyonlinelibrary.com]

This development is supported by PCA on plant macrofossil and sediment data (Figure 7). PCA of plant macrofossil data reveals a large overlap between individual polygons, indicating that the variance is not simply between individual sites (spatial), but within cores (temporal) as well (Figure 7a). Overall, 50.1% of the variance is explained by the first two PCA principal components, with PC1 representing 32.8% of the variance and PC2 17.3%. The plant macrofossil taxa *Carex*, *Potamogeton* and *Potentilla* correlate positively with PC1, while Eriophorum vaginatum, Betula glandulosa, Ledum decumbens and Vaccinium vitis‐idaea show negative correlation with PC1. The three first taxa indicate wet to submerged conditions. *Carex* is a typical wetland genus, indicating water‐saturated ground and even standing water from a few centimeters up to 1 m deep (cf Hannon and Gaillard,^56^ Cody^57^). *Carex* is especially abundant in low‐lying parts of ice‐wedge polygons and in shallow lake margins on the Yukon Coastal Plain.^31^, ^38^ Potentilla palustris is a wetland species commonly found around ice‐wedge troughs with standing water (our personal field observations) and generally on wet ground.^57^ *Potamogeton* is a true aquatic genus indicating water depths on the scale of decimeters to meters.^56^ Eriophorum vaginatum, Betula glandulosa, Ledum decumbens and Vaccinium vitis‐idaea are all typical of mesic conditions, as found on elevated, better drained surfaces in ice‐wedge polygons on the Yukon Coastal Plain.^31^ PCA of the sediment parameters TOC, C/N and δ^13^C illustrates that the main variance pattern in the core data is indeed associated with a hydrological gradient from submerged to well‐drained conditions (shallow lake to mesic ice‐wedge polygon, Figure 7b). The first two axes of PCA (PC1 and PC2) explain 84.2% of the variance in sediment parameters. TOC contents and C/N ratios are positively correlated with PC1 (51% explained variance), and mainly represent the difference between dwarf shrub/cottongrass peat, sedge peat and lake sediment. Stable carbon isotope composition is positively correlated with PC2 (33.2% explained variance). The peat samples are subdivided into submerged, wet and mesic polygon environments. The wet polygon samples show a wider range (larger dissimilarities) for TOC contents, but especially in δ^13^C (associated with PC2) than those of submerged polygons, but they overlap largely. Overall, organic‐rich lake sediment from shallow lakes is clearly separated from peat of submerged, wet and mesic ice‐wedge polygon microsites.

### 6.1 Komakuk Polygon development

#### 6.1.1 Center core

The center core exhibited two zones that we interpreted as lake sediments overlain by peat. In zone KP_c_1 (ca 1600–1450 cal years BP), fine‐grained sediments with TOC values around 10 wt.% indicated the presence of a lake environment rather than peat from an ice‐wedge polygon. While lake sediment in deeper parts of lakes in the region exhibits slightly lower TOC, lower C/N and higher δ^13^C,^55^, ^58^ the sediment we found resembled a transitional phase between lake drainage and ice‐wedge polygon initiation identified in a study from Herschel Island^12^ as well as a phase of low lake water level reconstructed in a study from a lake near Roland Bay.^38^ The plant macrofossil assemblage showed a mixture of mesic terrestrial, wet terrestrial, emergent and submerged aquatic taxa, indicating a highly productive shallow‐water environment in close proximity to drier terrestrial reaches. Modern satellite imagery showed the outline of a drained lake basin (Figure 2a). The studied polygon was situated in the marginal part of that former lake, which still existed during KP_c_1.

In KP_c_2 (at least 400 cal years BP to modern) peat established. Strong parallels in composition, plant macrofossil assemblage and thickness of this peat layer and the dated peat in KP_r_1 from the same ice‐wedge polygon suggested a similar age range for KP_c_2 (Figure 3). The base of the peat layer was dated to ca 2600 cal years BP, while the underlying lake sediment was much younger (median age 1461 cal years BP). This age inversion directly at the sedimentary facies break could have been caused by cryoturbation after lake drainage, when permafrost and periglacial features would have started to develop at the site.^59^, ^60^ This could not be ascertained, however, because no typical cryoturbated horizon was identified in the core. The older basal peat may have also been redeposited onto the newly drained lake surface, after which younger material started developing on top of the redeposited material. Redeposition of old organic material is the most common reason behind anomalous radiocarbon ages in permafrost.^61^, ^62^, ^63^, ^64^ The plant macrofossil mixture of mesic and wet terrestrial taxa (Table 2) indicated a low‐centered ice‐wedge polygon with no or very little standing water in the center.^31^ Toward the top of the core C/N increased. We suggest that this shift represented the conversion into an intermediate‐centered polygon, as litter with high C/N ratios is associated with mesic terrestrial plant taxa such as Betula glandulosa and Ledum decumbens,^65^ which are typically found on well‐drained sites within ice‐wedge polygons.^31^

#### 6.1.2 Rim core

The rim core featured a lower sediment facies typical of a low‐centered polygon superseded by a hiatus that we interpret as an erosion surface, and recent peat accumulation in the upper part of the core. KP_r_1 was dated to the two millennia around 5000 cal years BP, with an age inversion in the lowest sample (Figure 3, Table 1), suggesting a mid‐Holocene age range for KP_r_1. Fluctuating high TOC contents indicated either decomposing peat or varying input of inorganic material. Good preservation of plant macrofossils and narrow ranges in C/N and δ^13^C showed that organic material composition was stable, while organic matter contents varied, suggesting that peat decomposition played a minor role. The pattern was probably caused by varying input of fine‐grained sediment originating from sporadic disturbances. Plant macrofossils comprised mesic and wet terrestrials, and emergent and submerged aquatics. This assemblage indicated a highly structured wetland as found in low‐centered polygons with sufficiently deeply submerged centers to allow the growth of submerged *Potamogeton* (cf Hannon and Gaillard^56^). KP_r_1 was followed by a hiatus of ~5000 years, which coincided with a facies break. The polygon center core had a basal age of 1600 cal years BP, which placed the facies break in that core in a time slice lost from the rim core, indicating that lake sedimentation could have been active there at least after 1600 bp. We interpreted the upper surface of KP_r_1 and KP_c_1 as an erosion surface.

The peat in KP_r_2 developed within the past 300 years, as indicated by the results of radiocarbon dating (Table 1). Radiocarbon dates from this timeframe are generally ambiguous (deVries effect,^66^ Suess effect,^67^ atomic bomb effect^68^), limiting the temporal resolution for these depths. The zone was subdivided into KP_r_2A and KP_r_2B based on a shift in peat composition. Stable and very high TOC contents throughout KP_r_2 preclude increased decomposition of organic material as the cause of a pronounced increase in C/N ratios. Instead, plant macrofossils showed a shift from wet conditions in KP_r_2A to mesic conditions in KPr2B. Taxa typical of well‐drained elevated reaches within ice‐wedge polygons (Betula glandulosa, Ledum decumbens, Vaccinium vitis‐idaea, Eriophorum vaginatum)^31^ became dominant in subzone KP_r_2B, and their increasing abundance caused increased C/N ratios in the peat. We suggest that this vegetation shift accompanied the conversion of a low‐centered polygon into an intermediate‐centered polygon within the last 300 years.

The cores taken from Komakuk Polygon were spaced only 5 m apart, and reconstruction of polygon development at the site indicated the presence of a mid‐Holocene peatland, followed or interrupted by a phase of aquatic conditions in a lake margin environment, during which sediment was lost from the polygon rim by erosion and/or decomposition of organic material. Regular peat growth reinitiated during the last 300 years. Both cores showed shifts from aquatic vegetation to mesic ice‐wedge polygon vegetation, which is characterized by different habitats existing in close proximity. The conversion of a low‐centered polygon to an intermediate‐centered polygon occurred within the last 300 years, probably very recently.

### 6.2 Ptarmigan Polygon development

#### 6.2.1 Center core

The center core from Ptarmigan Polygon indicated lake sedimentation in the lower zone and peat accumulation typical of undisturbed low‐centered polygons in the upper zone. The lower boundary of this core probably corresponded to the upper surface of the glacial outwash plain, because coarse‐grained material typical of the unit described by Rampton^7^ stopped the corer at 88 cm depth (6380 cal years BP). In PP_c_1, only small amounts of plant material of mixed origin were present, and low and stable TOC and C/N values suggested a lake sedimentation environment. This interpretation is supported by the PCA of sediment parameters (Figure 7b), in which core parts we interpreted as shallow lake environments in Roland Polygon and Komakuk Polygon overlap nearly perfectly with those from the lower part of the Ptarmigan Polygon center core. Unlike in Komakuk Polygon, no aquatic plant macrofossils were found (Table 3), suggesting that the site was not located within the productive littoral zone of a lake, but in a slightly deeper, more central part, in which few terrestrial plant remains would be expected. In PP_c_2 (post‐bomb dates), sedge peat established, as evident from stable high TOC contents, consistently low C/N ratios (Figure 4) and Cyperaceae remains. These stable modern conditions in the center of the low‐centered polygon showed no indication of drier or wetter conditions or disturbances.

#### 6.2.2 Rim core

The polygon rim core consisted of one peaty sediment horizon. The core showed peat accumulation since 1100 cal years BP (Table 1, Figure 4). During that time, polygon rim conditions remained relatively stable, as indicated by stable TOC contents and grain size composition. A rise in C/N ratios was accompanied by an increase in dwarf shrub macrofossils toward the top of the core (Figure 4). This indicated drier conditions on the polygon rim in the recent past. Improved aeration in drier peat facilitates microbial activity and peat decomposition, and the gradual increase in δ^13^C values along the core could have been caused by increasing microbial utilization of carbon, which discriminates against the lighter ^12^C and thus leads to ^13^C enrichment.^69^

The combined information from both cores suggested the presence of a lake on the flat glacial outwash plain during the mid‐Holocene. In the course of the late Holocene, before 1100 cal years BP, that lake drained, and ice‐wedge polygons started to develop on the former lake floor. Peat initiation in Ptarmigan Polygon roughly fell within the timeframe given for the inundation of Workboat Passage by the Beaufort Sea, which was caused within the last 1600–600 years by sea level rise and which separated Herschel Island from the mainland.^70^, ^71^, ^72^ This event altered surface topography and hydrology, lowering the topographic gradient across the coastal plain, thus increasing surface water retention and facilitating the build up of peat in ice‐wedge polygons during at least 1100 years. In modern times, the analyzed polygon rim has experienced drying accompanied by carbon decomposition and an increase in shrubs.

### 6.3 Roland Polygon development

#### 6.3.1 Center core

Sediment composition and plant macrofossil assemblage in this core recorded the development from a shallow lake environment (~7000 cal years BP), to the initiation of a low‐centered polygon (~600 cal years BP), and subsequent gradual conversion to a high‐centered polygon (last century). During the period corresponding to zone RP_c_1 (~7000–600 cal years BP), a lake environment existed, as indicated by abundant occurrences of Charophyte oogonia, *Potamogeton* seeds and *Daphnia* ephippiae along with low TOC, low C/N, relatively high δ^13^C and a fine‐grained sediment texture (Figure 5, Table 4). Sediment composition and plant macrofossil assemblage resembled the productive lake margin or shallow lake environment already identified in KP_c_1 and in PP_c_1. The location of the studied polygon in the marginal part of a former lake can be inferred from modern satellite imagery (Figure 2c), in which both former lake extent and drainage path are visible.

During the time interval corresponding to RP_c_2 (~600 cal years BP to modern) peat established and aquatic taxa disappeared (Figure 5, Table 4). Over the centuries following peat initiation, a low‐centered polygon persisted at the site, as indicated by very high TOC contents, moderately high C/N ratios and relatively high δ^13^C accompanied by remains of mesic and wet terrestrial plant taxa in subzone RP_c_2A. In RP_c_2B, modern mesic conditions developed as a high‐centered polygon emerged. The lower boundary of zone RP_c_2B could not be more accurately dated, as the age range lay within the past 300 years, where radiocarbon dating is linked to large uncertainties (see above). In accordance with the available dates we suggest that the transition to drier conditions occurred within the last 100 years. Macroremains of plants were entirely from mesic taxa that were identified at the site during a vegetation survey in 2012 (eg, Eriophorum vaginatum, see Table 4, Wolter et al^31^). A sharp increase in C/N and a drop in δ^13^C indicated that carbon increasingly derived from terrestrial plant sources.^73^ TOC stayed very high and exceptionally stable, and thus we infer that the carbon signature did not present a decomposition signal, but an alteration in carbon source, toward more mesic plant taxa, particularly to an increase in the deciduous dwarf shrub Betula glandulosa.

#### 6.3.2 Margin core

The core showed peat of different genesis: the lower zone indicated a shallow submerged environment superseded by peat typical for low‐centered polygons and a hiatus we interpreted as an erosion surface, until in the upper zone peat formation was re‐initiated. The margin core from Roland Polygon was located only 4 m from the center core, and basal dates (~7000 cal years BP) matched the center core. RP_r_1A was, however, not made up of lake sediment but of peat from wet terrestrial plants, as indicated by very high TOC, relatively low C/N and high δ^13^C. The plant macrofossil record contained no aquatic plants. Instead, mesic terrestrials, large amounts of *Carex* seeds and some *Daphnia* resting eggs (ephippiae) were found (Figure 5, Table 4). The genus *Carex* contains semiaquatic species such as Carex aquatilis, which often dominates aquatic communities in tundra ponds associated with ice‐wedge polygonal terrain (eg, Bliss^74^). *Daphnia* is found in partly submerged areas around lakes or in ponds (eg, Gliwicz^75^). We suggest that during the mid‐Holocene an ice‐wedge polygon with a seasonally or permanently submerged pond existed in the shallow reaches of a lake as seen around modern lakes in the region (Figure 2a, c).

During the period corresponding to RP_r_1B drier conditions established, indicated by decreasing δ^13^C, rising C/N, decreasing amounts of *Carex* seeds, absence of aquatics and increasing dominance of mesic terrestrials (Figure 5, Table 4). The vegetation mosaic reflected typical moisture gradients found in ice‐wedge polygons in the region (eg, Wolter et al^31^). Radiocarbon ages in RP_r_1B ranged from ca 5000 cal years BP to dates within the last 300 years. The zone was capped by a distinct facies break, at which a hiatus of nearly 5000 years occurred within 3 cm of sediment (Table 1). This may have been caused by lateral displacement or decomposition of peat. We suggest that erosive action, rather than decomposition alone, caused the removal of material, as no signs of intensive decomposition were found in adjacent layers. A similar erosion surface was found in Komakuk Polygon, where it was most prominent in the polygon margin as well.

RP_r_2 comprised modern peat that formed within the last 300 years. Very high and uniform TOC contents indicated stable peat accumulation. The shift toward drier conditions that we saw in the polygon center core was repeated here, with C/N decreasing strongly and *Carex* disappearing. This supported evidence for conversion from a low‐centered polygon to a high‐centered polygon, probably as recently as 100 years ago.

Roland Polygon was located at the margins of a lake during the mid‐Holocene and at least seasonally submerged. At some point after lake drainage, erosive removal of material created a ~5000‐ year hiatus. The central part stabilized and has been accumulating peat in a low‐centered polygon since 600 cal years BP, and the margin followed during the last 300 years. The modern high‐centered polygon probably emerged during the last century.

### 6.4 Climate vs. geomorphic disturbances as drivers of change in ice‐wedge polygons

The prerequisites for ice‐wedge polygon development (waterlogged ground, permafrost, extreme ground‐penetrating cold during winter) are determined by climate and geomorphology. Ice‐wedge polygon initiation and conversion of low‐centered into high‐centered polygons is therefore strongly related to the dynamics of and the interplay between the two.

Investigations into radiocarbon dates have revealed broad climate‐induced simultaneous patterns of peatland initiation.^76^, ^77^ Strong seasonality and high summer temperatures have been suggested as drivers of intensive peatland formation during the Holocene thermal maximum in Alaska.^77^ Our study of mid‐ to late Holocene ice‐wedge polygon development found spatially heterogeneous peat formation in polygons around 7000 cal years BP (after the regional Holocene thermal maximum), under conditions much wetter than today (Figure 6). We found no climate‐induced peat initiation in the following millennia, when regional climatic patterns were largely stable. In the last millennium, however, evidence for lake drainage and peat accumulation in Komakuk Polygon and Roland Polygon during the regional Little Ice Age (ca ad 1600–1850; see^78^, ^79^, ^80^) suggests a climatic link.

Topographic evidence favors geomorphic causes for ice‐wedge polygon initiation on the Yukon Coastal Plain, where most polygon fields, including the ones we studied, are situated in drained thaw lake basins. The initiation of peat accumulation in Ptarmigan Polygon was probably linked to sea level rise in addition to lake drainage. The lake was probably already drained when Workboat Passage (Figure 1) was flooded 1600–600 years ago.^70^, ^71^, ^72^ The flooding caused a flattening of the relative topography in the area, with very low coastal bluffs (1–2 m). This increased water retention on land, facilitating ice‐wedge polygon development and peat growth. The link to local hydrological conditions as drivers of ice‐wedge polygon development has also been reported from Russian permafrost regions.^81^

The conversion of low‐centered polygons to high‐centered polygons is thought to be linked to internal self‐organization,^19^, ^21^ improved drainage (eg, Hussey and Michelson^82^) or melting of ice wedges.^4^ Shifts from aquatic to high‐moisture wetland vegetation and finally to mesic wetland vegetation were evident in our cores (Figures 3, 4, 5, 6, Tables 2, 3, 4). The conversion of low‐centered polygons to well‐drained forms occurred during the last 100–200 years in all polygons (Figures 3, 4, 5, 6, Tables 2, 3, 4). Komakuk Polygon switched from a low‐centered polygon with dwarf shrub growth on the rims to an intermediate‐centered polygon where dwarf shrubs had also established in the polygon center. Ptarmigan Polygon was the most stable over time, yet the polygon rim changed from Cyperaceae‐dominated to dwarf‐shrub‐dominated, indicating drying (Table 3). Roland Polygon showed complete development from a low‐centered to a high‐centered polygon. All three polygons showed signs of recent ice‐wedge degradation.^31^

The conversion of one polygon type to another may result from internal self‐organization through two main processes: lateral movement of material adjacent to ice wedges may widen ice‐wedge troughs and displace material toward the polygon center, where a mound establishes.^19^ Vegetation growth in polygon centers exceeding the upward growth of the surrounding ice wedges may also result in a well‐drained mound of peat surrounded by water‐filled trenches.^21^, ^83^ Both processes act on time‐scales of centuries to millennia, contrasting with the rapid conversions we found.

Improved drainage may result from a change in topographic gradient and thus in surface flow patterns, or from ice‐wedge degradation promoting drainage of polygon centers into the surrounding ice‐wedge troughs. The modern positions of Komakuk Polygon and Roland Polygon on elevated surfaces above lakeshore bluffs of several meters height (Figure 2a, c) indicate that drainage outweighs water input to these polygons, facilitating conversion to high‐centered polygons. The climate‐induced process of ice‐wedge degradation is also evident in the polygons^31^ and may be rapid: ice‐wedge degradation and establishment of drainage channels within a few decades have been reported from the Arctic Coastal Plain of Alaska,^4^, ^30^ the Eastern Canadian Arctic^20^ and Siberia.^84^

In the two studied ice‐wedge polygons that experienced conversion from low‐centered to intermediate‐centered (Komakuk Polygon) or high‐centered (Roland Polygon), both rim cores and one center core show a hiatus of at least 5000 years (Figure 6) caused by erosion of sedimentary material, indicating significant disturbance. Several processes might have caused material loss: lateral material displacement caused by ice wedge growth,^19^ increased runoff^4^ facilitating thermal erosion, erosion because of ice‐wedge degradation,^20^ peat decomposition linked to better aeration and higher temperatures (increased microbial activity),^21^ or fire.^85^ No disturbances in peat accumulation were indicated in low‐centered Ptarmigan Polygon (this study), nor in a low‐centered ice‐wedge polygon studied on Herschel Island,^12^ which showed undisturbed peat formation for the last 3000 years. The main Holocene and modern sources of disturbance on the Yukon Coastal Plain are mass wasting processes^86^, ^87^ linked to increased coastal erosion^88^ and thermokarst^64^ as well as thermal erosion.^89^ The question of whether disturbance triggered later drainage of the polygon centers and finally led to relief inversion cannot be answered at this stage, but will be worth investigating.

The changes we observed in peatland initiation and change from low‐centered to high‐centered were mostly caused by geomorphological change (sea‐level rise, tapping and draining of adjacent lakes, changes in drainage pathways across the landscape). In permafrost‐affected landscapes, climatic change may trigger widespread geomorphological change, especially where unconsolidated ice‐rich sediments dominate. Such climate‐induced geomorphological change may have locally variable impacts, but its frequency is likely to increase under climatic change. Regionally synchronized ice‐wedge polygon development requires a higher amplitude and seasonality of temperature and precipitation change than evident for the mid‐ to late Holocene. Our findings indicate that modern warming, however, may have triggered regional‐scale conversion from low‐centered polygons to high‐centered polygons. This process may rapidly initiate irreversible self‐enhancing erosion of ice‐wedge polygons.

Roland Polygon experienced stability for at least 2000 years during the mid‐Holocene (ca 7000–5000 cal years BP, Figure 5, Table 4), under considerably wetter conditions than today. The site was stable when a productive shallow lake area existed directly adjacent to or overlapping the partly submerged ice‐wedge polygon. Ptarmigan Polygon had been stable at least from 1100 cal years BP until recent drying and shrub expansion into the polygon. The protected and low coast along Workboat Passage probably facilitated ice‐wedge polygon stability by minimizing drainage. Stable conditions over millennia have been reported from an ice‐wedge polygon on Herschel Island.^12^ Its position in a depression between rolling hills probably provided the polygon with excess surface moisture, outweighing drainage through an outlet channel down the coastal bluffs.

Late Holocene climatic conditions were relatively stable on the Yukon Coastal Plain compared with high‐amplitude oscillations at the Pleistocene–Holocene transition and during the early Holocene (Figure 6). The main climatic change was related to increasing proximity to the sea, causing lower summer temperatures^5^, ^90^ and increased summer precipitation,^55^ both likely to stabilize ice‐wedge polygons. Our results indicate that a non‐negative water balance was the main factor promoting stability during low‐amplitude climatic fluctuations. Stable low‐centered polygons prevail when continued moisture supply outweighs drainage and evapotranspiration. In contrast, decreasing moisture supply from the surrounding landscape or increasing drainage caused by geomorphological processes such as coastal erosion, thermal erosion or thermokarst trigger conversion into high‐centered polygons.

## 7 CONCLUSIONS

We reconstructed mid‐Holocene and Holocene landscape features in coastal lowland tundra on the Yukon Coastal Plain. We traced the development of shallow lakes to low‐centered ice‐wedge polygons and subsequently to high‐centered polygons. During the mid‐Holocene, the studied sites contained shallow lakes or submerged polygon centers and paleobotanic data generally reflect wetter conditions than today. An erosional hiatus of ca 5000 years indicates disturbance and erosion in high‐ and intermediate‐centered polygons which formed later on. In recent decades, ice‐wedge polygons on the Yukon Coastal Plain experienced degradation and drying through warming‐induced geomorphological change. In our study, the main driver of (i) ice‐wedge polygon initiation was lake drainage. The main driver triggering (ii) conversion of low‐centered polygons to high‐centered polygons was improved drainage through ice‐wedge degradation and changes in the local topographic gradient. By contrast, we found that stable conditions prevailed for millennia during the late Holocene in ice‐wedge polygons under low‐amplitude climatic change as long as a non‐negative water balance was maintained in the polygon field. Hence, geomorphic disturbance was the main driver of locally variable ice‐wedge polygon dynamics in periods of low climatic forcing. Extreme climatic change triggered simultaneous developments, such as widespread peat initiation during the Holocene Thermal Maximum. Modeling of the development of polygon fields through time must thus focus on temperature constraints as well as landscape water balance and flow paths.
